# Identification of apoptosis-related gene signatures as potential biomarkers for differentiating active from latent tuberculosis via bioinformatics analysis

**DOI:** 10.3389/fcimb.2024.1285493

**Published:** 2024-01-19

**Authors:** Xiaoting Dai, Litian Zhou, Xiaopu He, Jie Hua, Liang Chen, Yingying Lu

**Affiliations:** ^1^ Department of Infectious Diseases, Nanjing Lishui People’s Hospital, Zhongda Hospital Lishui Branch, Southeast University, Nanjing, China; ^2^ Department of Neurosugery, Nanjing Lishui People’s Hospital, Zhongda Hospital Lishui Branch, Southeast University, Nanjing, China; ^3^ Department of Geriatric Gastroenterology, The First Affiliated Hospital of Nanjing Medical University, Nanjing, China; ^4^ Department of Gastroenterology, The First Affiliated Hospital of Nanjing Medical University, Nanjing, China; ^5^ Department of Clinical Laboratory, Seventh People’s Hospital of Shanghai University of Traditional Chinese Medicine, Shanghai, China; ^6^ Department of Clinical Laboratory, Shanghai Pudong New Area People’s Hospital, Shanghai, China

**Keywords:** *Mycobacterium tuberculosis*, active tuberculosis, latent tuberculosis infection, apoptosis, biomarkers

## Abstract

**Background:**

Apoptosis is associated with the pathogenesis of *Mycobacterium tuberculosis* infection. This study aims to identify apoptosis-related genes as biomarkers for differentiating active tuberculosis (ATB) from latent tuberculosis infection (LTBI).

**Methods:**

The tuberculosis (TB) datasets (GSE19491, GSE62525, and GSE28623) were downloaded from the Gene Expression Omnibus (GEO) database. The diagnostic biomarkers differentiating ATB from LTBI were identified by combining the data of protein-protein interaction network, differentially expressed gene, Weighted Gene Co-Expression Network Analysis (WGCNA), and receiver operating characteristic (ROC) analyses. Machine learning algorithms were employed to validate the diagnostic ability of the biomarkers. Enrichment analysis for biomarkers was established, and potential drugs were predicted. The association between biomarkers and N6-methyladenosine (m6A) or 5-methylated cytosine (m5C) regulators was evaluated.

**Results:**

Six biomarkers including *CASP1, TNFSF10, CASP4, CASP5, IFI16*, and *ATF3* were obtained for differentiating ATB from LTBI. They showed strong diagnostic performances, with area under ROC (AUC) values > 0.7. Enrichment analysis demonstrated that the biomarkers were involved in immune and inflammation responses. Furthermore, 24 drugs, including progesterone and emricasan, were predicted. The correlation analysis revealed that biomarkers were positively correlated with most m6A or m5C regulators.

**Conclusion:**

The six ARGs can serve as effective biomarkers differentiating ATB from LTBI and provide insight into the pathogenesis of *Mycobacterium tuberculosis* infection.

## 1 Introduction

*Mycobacterium tuberculosis* (MTB), the causative agent of tuberculosis (TB), represents one of the most lethal pathogen-related infections globally. According to the Global Tuberculosis Report 2022, about 10.6 million new TB cases have been diagnosed and 1.6 million deaths have occurred in 2021 (Bagcchi, 2023). Of 2 - 3 billion MTB-infected individuals, about 5-15% develop TB, with a higher risk among young children (Carvalho et al., 2018). The mechanisms and factors associated with transitioning from latent TB (LTBI) to active TB (ATB) infection remain undetermined, and their clinical differentiation is challenging despite their importance in prognosis and appropriate treatments. Interferon (IFN) γ release assay (IGRA) and tuberculin skin test (TST) are commonly employed for TB diagnosis. However, neither can differentiate between ATB and LTBI, and they might yield non-reactive results in TB patients with immune suppression or malnutrition (Pai and Behr, 2016). Furthermore, TST often indicates false positive results in Bacillus Calmette–Guérin vaccinated individuals (Pai and Behr, 2016). Atypical manifestations often delay and complicate TB diagnosis in many cases. Therefore, it is critical to identify alternative biomarkers to distinguish ATB from LTBI.

The death of host cells significantly influences the transition of LTBI to ATB (Ni Cheallaigh et al., 2011; Nisa et al., 2022). Some gene signatures have indicated the potential pathogenesis and can be novel biomarkers for distinguishing the two TB states (Deretic et al., 2006; Ni Cheallaigh et al., 2011). Apoptosis is a highly regulated cellular mechanism that alleviates inflammation and injury by containing dying cells’ disintegrated cytoplasmic and nuclear contents in membrane-bound vesicles (apoptotic bodies) engulfed by other phagocytes in efferocytosis (Nisa et al., 2022). The apoptotic process includes cell shrinkage, nuclear fragmentation, chromatin condensation, and outer cell membrane blebbing, forming an apoptotic body. Internucleosomal boundaries cleave chromosomal DNA, evidenced by DNA bands on gel electrophoresis. Phosphatidylserine is a membrane component on the viable cells’ cytosolic side, and in apoptotic cells, the enzyme flippase translocates it to the outward-facing surface (Nisa et al., 2022). Apoptosis cleans the intracellular bacteria and activates the hosts’ adaptive immune response. As per histopathological analysis, granulomatous tissue surrounding the centralized caseation areas has numerous cells, suggesting that apoptosis can protect from MTB by limiting bacterial dissemination. Interestingly, avirulent MTB strains induce apoptosis, while virulent strains suppress it, benefiting the pathogens’ dissemination (Lam et al., 2017).

This study aims to identify apoptosis-related gene (ARG) signatures for differentiating ATB and LTBI, explore their association with the immune cell populations, N6-methyladenosine (m6A)- and 5-methylated cytosine (m5C) regulators, and predict drugs via the bioinformatics approach.

## 2 Materials and methods

### 2.1 Data extraction

This study included mRNA expression data obtained from the NCBI-GEO (http://www.ncbi.nlm.nih.gov/geo); blood samples of patients who (1) were > 15 years old, (2) gave samples before the antimycobacterial regimen started, and (3) did not have human immunodeficiency virus (HIV) infection. ATB patients were diagnosed based on confirmed isolation and MTB culturing of respiratory samples, negative MTB cultures, TB-related clinical symptoms, and radiological and clinical data. LTBII was diagnosed based on confirmed contact with individuals with positive TB smear results and TST or IGRA data without any clinical or radiological signs of ATB on follow-up.

Three microarray databases (GSE19491, GSE62525, and GSE2623) were extracted as per the above criteria. The training set GSE19491 (Platform-GPL6947) comprised 54 ATB and 69 LTBI blood samples, and the validation sets included GSE62525 (Platform-GPL16951; 14 ATB and 14 LTBI peripheral blood mononuclear cell (PBMC) samples) and GSE28623 (Platform-GPL4133; 46 ATB and 25 LTBI whole blood samples). Furthermore, 680 ARGs were acquired from the Kyoto Encyclopedia of Genes and Genomes (KEGG) and Gene Ontology (GO) datasets in Gene Set Enrichment Analysis (GSEA) (https://www.gsea-msigdb.org/gsea/index.jsp).

### 2.2 Immune cell infiltration assessment

In GSE19491 of both groups, the abundance of 28 infiltrating immune cells was assessed via single sample GSEA (ssGSEA) using the “GSVA” R package (v 1.42.0). Moreover, the differences between LTBI and ATB groupd were compared using the Wilcoxon test. The infiltrating immune cells with considerable differences were utilized for further study (*p < 0.05*).

### 2.3 Weighted gene co-expression network generation

Since several studies have shown that apoptosis is inextricably linked to immune cell infiltration ((Liu et al., 2023). Therefore, immune cell-related genes (ICRGs) were used for screening apoptosis-related biomarkers for ATB. The ICRGs in this study were obtained by WGCNA analysis. Each infiltrating immune cell that significantly differed in abundance between ATB and LTBI was considered a trait. The “WGCNA” R package (v 1.70-3) was employed for WGCNA. The samples were clustered to remove outliers. A soft threshold (β) determination was performed to ensure that gene interactions conform to the maximum extent of scale-free distribution. The similarity among genes was calculated according to the adjacency. The gene dendrogram, including different modules, was developed based on dissimilarities. The relevance between modules and traits was assessed using the Pearson correlation analysis, and those with relatively high correlations were selected. Furthermore, modules’ genes were combined and labeled immune cell-related genes (ICRGs).

### 2.4 Differentially expressed ARGs and biomarkers Screening

The differentially expressed genes (DEGs) between ATB and LTBI groups were identified in GSE19491 via the “limma” R package (v 3.48.3) (adjusted *P* < 0.05). ARGs, ICRGs, and DEGs were intersected to acquire DE-ARGs via the “Veen Diagram” R package (v 1.6.20). Furthermore, a chromosomal localization analysis was performed for DE-ARGs by the “OmicCricos” R package (v 1.32.0). The GO and KEGG-based DE-ARGs enrichment analysis was performed via the “clusterProfiler” R package (v 4.0.2) (adjusted *P* < 0.05).

The protein-protein interaction (PPI) (medium confidence > 0.4) network for DE-ARGs was generated while employing the STRING database (http://string.embl.de/). Four algorithms (Degree, MNC, MCC, and EPC) in cytohubba identified the top 10 hub genes in the PPI network and were intersected to acquire common hub genes (CHG). Subsequently, in GSE19491, the CHGs expression in the ATB and LTBI groups was analyzed via the Wilcoxon test. Their diagnostic value for ATB was evaluated by Receiver Operating Characteristic (ROC) curves via the “pROC” R package (v 1.17.0.1). CHGs with an area under the curve (AUC) > 0.7 were considered biomarkers. The Spearman algorithm was employed to evaluate the correlation between biomarkers using the “corrplot” R package (v 0.90).

### 2.5 Assessment of the diagnostic value of biomarkers for ATB and qRT-PCR

According to the biomarkers’ expression, separate diagnostic models were developed via Random Forest (RF) (“randomforest” R package, v 4.7-1), Least Absolute Shrinkage and Selection Operator (LASSO) (“glmnet” R package, v 4.1-3), and logistic regression (“stat” R package). The diagnostic ability of the biomarkers was assessed via the ROC, and validation sets were validated.

Total RNA content was extracted from 20 paired ATB (female/male 10:10, age: 36.8 ± 10.3 yrs) and LTBI samples (female/male 10:10, age: 35.7 ± 7.4 yrs) using TRIzol kit (Life Technologies, Carlsbad, CA, USA) following the given instructions. All patients were HIV(-) and were not on any antimycobacterial treatment. For reverse transcription, PrimeScript RT Master Mix (Takara in Tokyo, Japan) was utilized, and the acquired cDNA was amplified using the ABI 7700 system (Applied Biosystems in CA, USA). β-lactin was employed as housekeeping control to normalize relative expression levels, assessed by the 2-ΔΔCt method. **Supplementary File 1** depicts the primer sequences employed for qRT-PCR.

### 2.6 Functional enrichment analysis

The biomarkers’ gene function similarity was measured by the “GOSemSim” R package. To explore biomarker-related functions and signaling pathways, GSE19491 samples were divided into high and low-expression groups based on biomarker expression’s median, followed by differential analysis. All genes were ranked according to logFC, and the ‘C2: KEGG gene sets’ were used as reference.

### 2.7 Drug prediction

The Drug-Gene Interaction Database (DGIdb, https://dgidb.org/) is a database of drug-gene interactions that provides information on the association of genes with their known or potential drugs. In this study, the drugs targeting biomarkers were predicted using the DGIdb database, and the network was visualized by the Cytoscape software (v 3.8.2).

### 2.8 Correlation analysis

The correlation between biomarkers and immune cells that significantly differed in infiltration abundance between ATB and LTBI was evaluated via the Spearman algorithm. The m6A and m5C are strongly associated with various diseases; therefore, the association between them and the biomarkers was analyzed. In GSE19491, the expression of 17 m6A regulators and 20 m5C regulators was identified, and the differences between the two groups were compared. The relevance between biomarkers and m6A or m5C regulators was then computed via the Spearman algorithm in the “psych” R package.

### 2.9 Statistical analysis

For statistical measurements, the R software (v4.1.0, https://www.r-project.org/) was utilized, and the inter-group differences were analyzed via the Wilcox test. *P <0.05* was considered statistically significant.

## 3 Results

### 3.1 A total of 289 ICRGs were obtained

**Figure 1:**
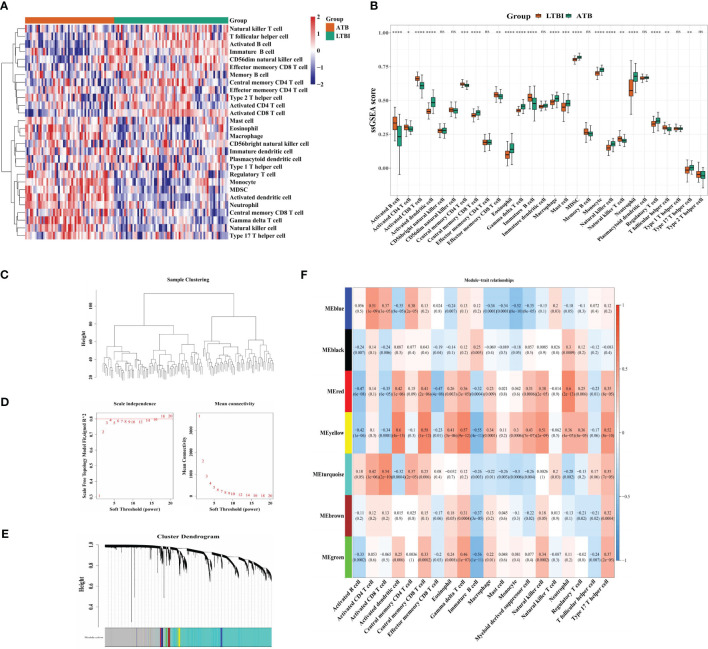
Identification of 289 immune cell-related genes. **(A)** Heatmap and **(B)** Boxplots indicating immune cell infiltration in LTBI and ATB samples. **(C)** Clustering of module eigengenes. **(D)** The selection of soft threshold power. **(E)** Gene dendrograms. **(F)** Correlation analysis of immune cells and module eigengenes. Row = module, and column = immune cell. *p < 0.05, **p < 0.01, ***p < 0.001; ****p < 0.0001; ns: not significant.

A heatmap was used to display the abundance of 28 infiltrating immune cells in both the ATB and LTBI groups (**Figure 1A**). Of these, 20 immune cells indicated a significant difference. The lymphocyte-related adaptive immune responses (activated B cells and CD 8+ and CD4+ T cells, etc.) were suppressed, whereas that of myeloid and inflammatory cells (macrophages, neutrophils, and monocytes) were increased (**Figure 1B**). Therefore, these 20 immune cells were used as traits to perform WGCNA. No outlier samples were observed in the training set (**Figure 1C**). At β = 18, the mean connectivity converged to 0 (**Figure 1D**). Altogether, seven modules were characterized, each with a unique color (**Figure 1E, F**). The MEyellow and MEred modules were highly correlated with most traits, such as monocytes, neutrophils, and activated CD8 T, B, and dendritic cells (DCs), etc. (**Figure 1F**), and were selected for further studies. There were 289 ICRGs for subsequent analysis.

### 3.2 There were 20 DE-ARGs between the ATB and LTBI groups

**Figure 2:**
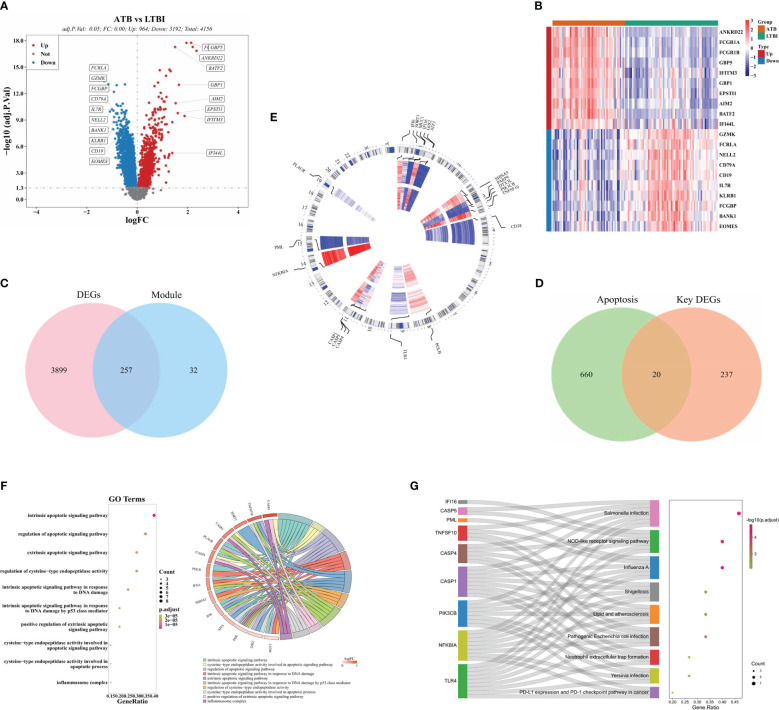
Identification of 20 DE-ARGs between LTBI and ATB groups. **(A)** A volcanic plot of DEGs between ATB and LTBI groups. **(B)** A heatmap of the top 20 DEGs between ATB and LTBI groups. Red = upregulated genes, and blue = downregulated genes. **(C)** Venn diagram indicating where the DEGs and ICRGs overlap and **(D)** where the key DEGs and ARGs overlap. **(E)** Chromosome localization of the 20 DE-ARGs. **(F)**. GO and **(G)** KEGG enrichment of the 20 DE-ARGs.

A total of 4,156 DEGs (964 upregulated and 3,192 down-regulated) were identified in the ATB and LTBI groups (**Figure 2A, B**). After intersection, 20 DE-ARGs were acquired (**Figure 2C, D**). The chromosome localization showed that in ATB and LTBI samples, six DE-ARGs (*IFI6, SORT1, MUC1, IFI16, G0S2*, and *ATF3*) were located on chromosome 1, five (*SHISA5, PARP9, DTX3L, PIK3CB*, and *TNFSF10*) on chromosome 3, three (*CASP1, CASP5*, and *CSAP4*) on chromosome 11 (**Figure 2E**), and six (*CD38, POLB, TLR4, NFKBIA, PML*, and *PLAUR*) on chromosomes 4, 8, 9, 14, 15, and 19, respectively (**Figure 2E**).

GO analysis revealed significant enrichment of 186 GO items including 163 biological processes, 2 cellular components, and 21 molecular functions (**Supplementary File 2**). These enrichments included ‘intrinsic apoptotic signaling pathway in response to DNA damage,’ ‘apoptotic signaling pathway modulation,’ and ‘intrinsic apoptotic signaling pathway,’ (**Figure 2F**). KEGG analysis revealed 29 enriched pathways (**Supplementary File 3**), including the DE-ARGs involved in ‘Salmonella infection,’ ‘Influenza A,’ and ‘NOD-like receptor signaling pathway.’ (**Figure 2G**).

### 3.3 There were six biomarkers with high ATB diagnostic values

**Figure 3:**
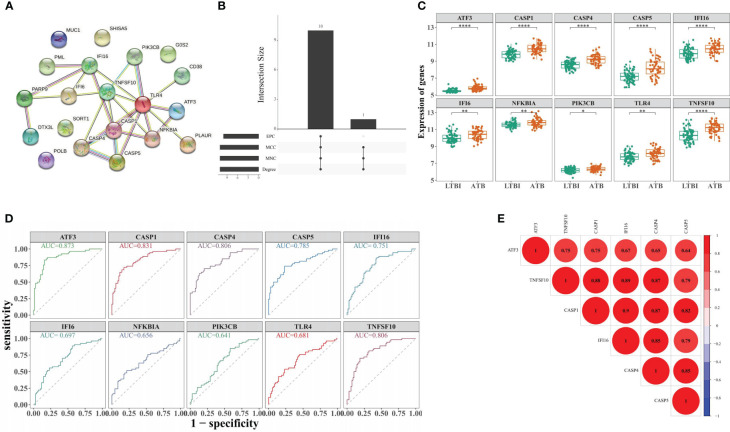
Identification of biomarker genes distinguishing ATB from LTBI. **(A)** Construction of the PPI network for DE-ARGs. **(B)** Screening of 10 CHG through four algorithms. **(C)** The expression levels of 10 CHG in ATB and LTBI samples. **(D)** The 10 CHG diagnostic values were acquired using the ROC analysis in the test set. **(E)** The correlation analysis of six biomarker genes. *p < 0.05, **p < 0.01, ****p < 0.0001.

The PPI network for DE-ARGs indicated that *TLR4* intersected with 9 proteins, including *PIK3CB, CASP1,* and *NFKBIA* (**Figure 3A**). Through four algorithms, 10 CHG, including *TLR4, CASP1, TNFSF10, CASP4, NFKBIA, CASP5, IFI16, IFI6, PIK3CB*, and *ATF3* were acquired (**Supplementary File 4** and **Figure 3B**). In the training set, these genes were all highly expressed in the ATB groups (**Figure 3C**). Moreover, CASP1, TNFSF10, CASP4, CASP5, IFI16, and ATF3 demonstrated AUC values more 0.7, (**Figure 3D**), qualifying them as selected biomarkers. The correlation analysis suggested a positive association among these biomarkers, where *IFI16* was most positively linked with *CASP1* (r = 0.90) (**Figure 3E**).

**Figure 4:**
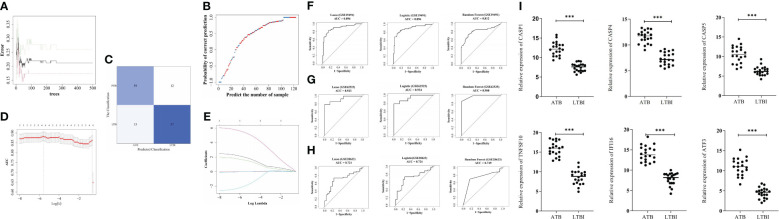
Construction of diagnostic models by machine learning algorithms. **(A)** The best decision tree in the random forest (RF) model was identified as 39. **(B)** The probability of correct prediction. **(C)** Confusion matrix of the RF model. **(D)** The coefficient of hub genes in Least Absolute Shrinkage and Selection Operator (LASSO) analysis. **(E)** Identification of the optimal penalization coefficient (λ) in the LASSO model by 10-fold cross-validation and the minimum criterion. ROC analysis of GSE19491 **(F)**, GSE62525 **(G)**, and GSE28623 **(H)**. The six biomarker genes mRNA levels in blood samples from 10 pairs of ATB and LTBI patients **(I)**. ***p < 0.001.

To investigate the biomarkers’ ATB diagnostic ability, the RF, logistic regression, and LASSO models were established (**Figure 4A-E**). In the training set, the three models indicated > 0.8 AUC values (**Figure 4F**), whereas in GSE62525, the AUC values were >0.98, >0.934, and > 0.923, respectively (**Figure 4G**). In GSE28623, the three models indicated > 0.7 AUC values (**Figure 4H**), indicating higher ATB diagnostic values of biomarkers.

### 3.4 qRT-PCR

The mRNA levels of *CASP1, TNFSF10, CASP4, CASP5, IFI16*, and *ATF3* in blood samples of ATB and LTBI patients were confirmed using qRT-PCR. The results revealed significantly higher expression of these six genes in ATB patients compared to LTBI patients (**Figure 4I**).

### 3.5 Enrichment analysis and drug rediction

**Figure 5:**
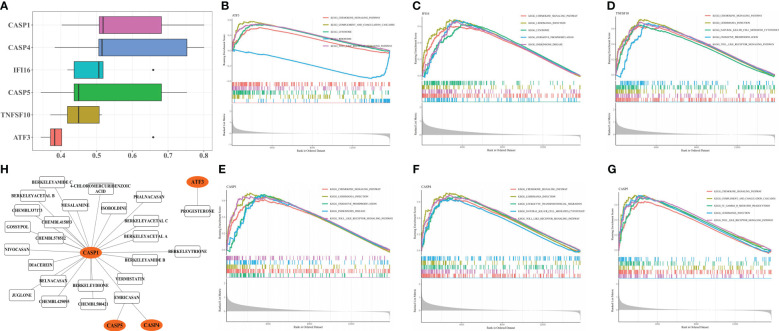
Enrichment Analysis and Drug Prediction of six biomarker genes. Functional similarity analysis of six biomarker genes **(A)**. Single-gene GSEA-KEGG pathway analysis of ATF3 **(B)**, IFI16 **(C)**, TNFSF10 **(D)**, CASP1 **(E)**, CASP4 **(F)** and CASP5 **(G)**. Construction of biomarker genes-drug network **(H)**.

The functional similarity analysis revealed a higher score of *CASP1* and *CASP4*, demonstrating similarities between their functions (**Figure 5A**). The top 5 KEGG pathways were acquired from the GSEA, and all biomarkers were involved in the ‘chemokine signaling pathway’ (**Figure 5B-G**). *IFI16, TNFSF10, CASP1, CASP4*, and *CASP5* were enriched in ‘leishmania infection’ (**Figure 5C-G**). Furthermore, *ATF3, TNFSF10, CASP1, CASP4*, and *CASP5* were enriched in a ‘TOLL-like receptor signaling pathway’ (**Figure 5B, D-G**), and *ATF3* and *CASP5* in ‘complement and coagulation cascades’ (**Figure 5B, G**).

The biomarker-drug network included 4 biomarkers (*CASP1, CASP5, CASP4*, and *ATF3*) and 24 drugs (**Figure 5H**). For *ATF3*, only progesterone was predicted, whereas *CASP1, CASP5*, and *CASP4* interacted with emricasan.

### 3.6 Association between biomarkers and immune cells or m6A or m5C-related genes

**Figure 6:**
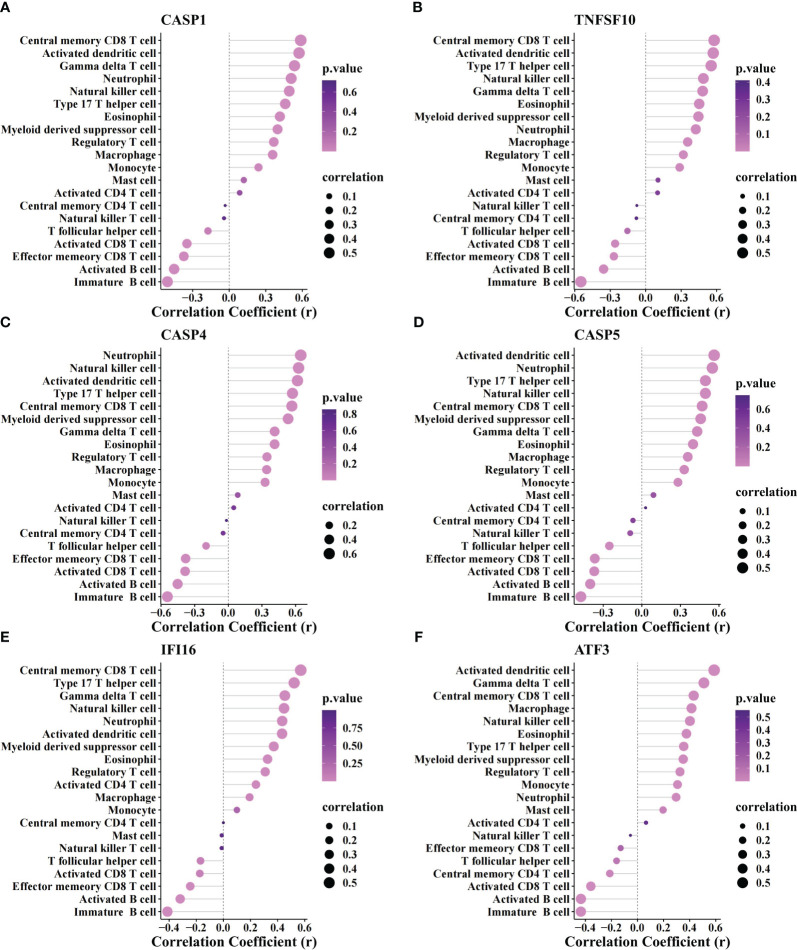
Correlation analysis between biomarker genes and immune cells. The association of immune cells with CASP1 **(A)**, TNFSF10 **(B)**, CASP4 **(C)**, CASP5 **(D)**, IFI16 **(E)**, and ATF3 **(F)**.

The correlation between biomarkers and immune cells revealed a positive association of all biomarkers with myeloid and inflammatory cells (neutrophils, activated DCs, macrophages, myeloid-derived suppressor cells, monocytes, etc.) and a markedly negative association with activated CD 8+ T and B cells (**Figure 6**).

**Figure 7:**
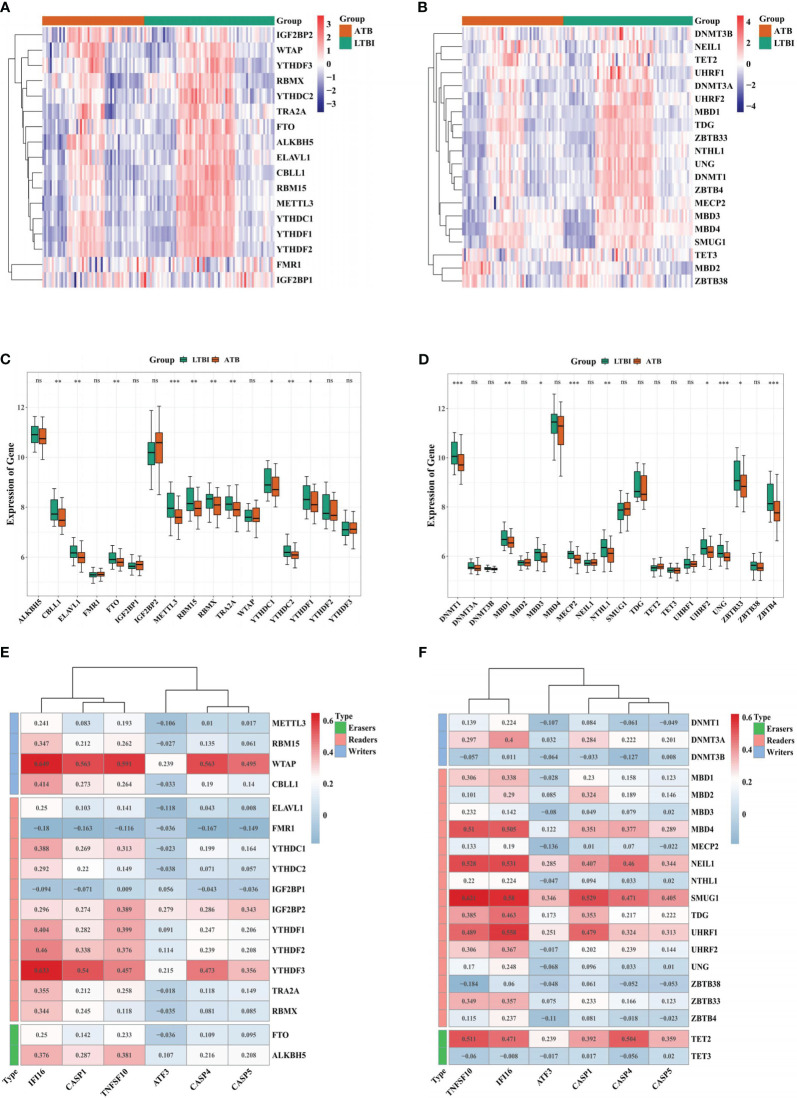
Correlation analysis between biomarker genes and m6A or m5C-related genes. Heatmap of the expression of 17 m6A-related genes **(A)** and 20 m5C-related genes **(B)** in ATB and LTBI samples. Boxplot of the expression of 17 m6A-related genes **(C)** and 20 m5C-related genes **(D)** in ATB and LTBI samples. The correlation analysis of 17 m6A-related genes **(E)** and 20 m5C-related genes **(F)** with six biomarker ARGs. *p < 0.05, **p < 0.01, ***p < 0.001. ns: not significant.

The expression of 17 m6A and 20 m5C-related genes in the two groups was depicted via heatmaps (**Figure 7A, B**). There were 10 m6A (*YTHDC1, YTHDF1, CBLL1, ELAVL1, FTO, RBM15, RBMX, TRA2A, YTHDC2*, and *METTL3)* (**Figure 7C**) and 9 m5C (*MBD3, UHRF2, ZBTB33, MBD1, NTHL1, DNMT1, MECP2, UNG*, and *ZBTB4*) related DEGs in ATB and LTBI groups, (**Figure 7D**). The correlation analysis indicated that WTAP had a significant positive association with IFI16 (r = 0.650, P = 4.62E-16), whereas *FMR1* had a significant negative association with *IFI16* (r = -0.180, P = 0.046) (**Figure 7E**). *TNFSF10* was positively relevant to *SMUG1* (r = 0.621, P = 1.83E-14) while negatively associated with *ZBTB38* (r = -0.184, P = 0.041) (**Figure 7F**).

## 4 Discussion

Despite recent advances in treatment and diagnosis, TB remains a leading infectious disease causing significant comorbidities and mortality globally (Carvalho et al., 2018; Bagcchi, 2023). Transcriptome investigations have identified the association of several genes and their expression patterns with TB pathogenesis. Host cell death manipulates MTB infection by controlling and restricting its dissemination. Increased necrotic death allows MTB to disseminate to proximal cells upon lytic death. Apoptosis, a regulated cell death process, is regarded as a defensive cellular mechanism against intracellular MTB during infection (Deretic et al., 2006; Ni Cheallaigh et al., 2011; Nisa et al., 2022).

Host immune response is the major mechanism against MTB infection (Mayer-Barber and Barber, 2015; Lu et al., 2021; Rijnink et al., 2021). Immune cell infiltration assessment revealed enhanced inflammatory and myeloid cell levels (DCs, monocytes, and neutrophils) in ATB patients than in LTBI patients, whereas adaptive immunity cells (active CD8 T and B cells) indicated reduced expression. The primary mechanism against chronic MTB infection is adaptive immunity, contributing to persistent LTBI. LTBI-diagnosed individuals have indicated MHC-I-restricted CD8+T cells in the blood and bronchoalveolar lavage fluid, which respond to MTB (Rijnink et al., 2021). B cells also contribute to the anti-TB immune processes, acting in germinal centers to generate innate and adaptive immunity-regulated antibodies, increasing the antigen presentation to T cells, and secreting cytokines to support T cell responses (Lu et al., 2021). These T cells and antibodies influence granuloma formation, thereby affecting the progression of MTB infection. In ATB patients, host responses against bacteria and related tissue injury further enhance inflammatory responses, inducing inflammatory cell (macrophages, DCs, neutrophils, and monocytes) proliferation (Mayer-Barber and Barber, 2015).

This study identified six gene signatures (*CASP1, CASP4, CASP5, TNFSF10, IFI16*, and *ATF3*) associated with suppressive adaptive immune and increased inflammation responses as biomarkers for differentiating ATB from LTBI. The caspase-cascade system is essentially associated with intracellular apoptotic signals’ induction, transduction, and amplification (Fan et al., 2005). The caspase family is categorized into two subgroups: those closely linked with ICE/caspase-1 (caspase-1, -4, -5), activated during inflammatory responses, and those stimulated during apoptosis (caspase-2, -3, -6, -10) (Fan et al., 2005). Caspase-1 processes the proinflammatory cytokine IL-1β and IL-18 in human monocytes and macrophages (Fan et al., 2005). In humans, caspase-4 and -5 activate caspase-1 and produce IL-1β (Fan et al., 2005). CASP-1-knockdown mice had reduced IL-1 and IL-18 levels; however, they did not indicate any overt defects in apoptosis regulation, strongly suggesting that this caspase is predominantly associated with cytokine maturation rather than cell death control (Van Opdenbosch and Lamkanfi, 2019). Increased active IL-1β generation and release can extensively and irreparably damage tissues. Further, caspase-1, 4, and 5 can mediate inflammasome (Vigano et al., 2015). Aberrant NLRP3 activation causes several types of cell death such as pyroptosis, necroptosis, and ferroptosis. This leads to compromised outer cell membrane integrity and the release of cytoplasmic and nuclear contents into the extracellular space. These events increased inflammatory responses and facilitate the dissemination of MTB (Vigano et al., 2015).

Tumor necrosis factor α-associated apoptosis-mediated ligand (TRAIL/TNFSF10) belongs to the TNF superfamily and induces apoptosis by interacting with the TRAILR1/death receptor 4 (DR4) and TRAILR2/DR5 (Cardoso Alves et al., 2021). TNFSF10 is expressed in various tissues and cells, with a primary localization on the cell surface of immune cells. The fraction of MTB cell wall can release soluble TNFSF10 from neutrophils (Kuribayashi et al., 2008). Cytotoxic T and natural killer cells induce target cells’ apoptosis via death receptor ligands (e.g., FasL and TNFSF10), which is crucial for controlling intracellular pathogenic infections (Kuribayashi et al., 2008). *In-vitro*, TB patient’s CD8+ T cells recognized HLA-E-binding MTB peptides and produced type 2 cytokines, which mediate TNFSF10-dependent cytolytic and microbicidal activity against MTB-infected cells (Caccamo et al., 2015). Therefore, the TNFSF10 upregulation is an anti-MTB mechanism. Furthermore, Manna et al. (La Manna et al., 2018) found that the serum TNFSF10 was significantly higher in ATB patients, consistent with this investigation.

The IFN-γ–inducible factor 16 (IFI16), a hematopoietic IFN-inducible nuclear antigen with a 200 amino acid repeat family, is detected in lymphocytes’ nuclei in the spleen, thymus, lymph nodes, and epithelial cells of these tissues (Choubey and Panchanathan, 2016). After the hosts’ innate immunity over activation, the IFI16 stimulator of IFN genes (STING) is dependent on IFN-1, which mediates TB pathogenesis (Choubey and Panchanathan, 2016). In MTB-infected mice, increased IFN-1 expression was found to be detrimental to survival and correlated with a reduction in Th1 immunity (Akter et al., 2022). Whole blood RNA signatures with increased IFN-1 signaling can determine individuals who can develop active disease (de Sa et al., 2022). IFI16 is in the cytosol during macrophage MTB infection, and MTB DNA stimulates the cytosolic surveillance mechanism. Furthermore, in the DNA-damaged cells, the IFI16 protein expression is induced by p53 activation and IFN-signaling. IFI16 enhances p53-mediated cell growth and regulates apoptosis, suggesting its involvement in anti-TB infection via apoptosis (Li et al., 2021b).

Activating transcription factor 3 (ATF3), a member of ATF/cyclic adenosine monophosphate (AMP) response element-binding family of TFs, is a pro-apoptotic protein that induces airway epithelial apoptosis via transcriptional regulation of DR5 and Bcl-xL (Du et al., 2022). *In-vitro*, ATF3 can change TNF α-dependent cell death mode from apoptosis to necroptosis (Du et al., 2022). It can also inhibit pro-inflammatory cytokine levels and stimulate the transcription of several inflammatory genes (TNF‐α, IL‐6, IL-12, etc.) related to increased inflammatory cells (Peace and O’Neill, 2022). Furthermore, ATF3 is crucial for modulating IFN‐β production downstream of innate immune receptors, suppressing cellular immunity against *MTB* infection (Peace and O’Neill, 2022).

Moreover, 24 potential drugs targeting four biomarker genes were screened from the DSigDB database. Many conditioned medium experiments indicated that stimulating progesterone receptors leads to increased ATF3 expression, which is associated with upregulated inflammation genes (TNF‐α and IFN-β) and cellular immunity inhibition (Shah et al., 2019; Motomura et al., 2023). Elevated progesterone levels are associated with diminished activities of T cells and natural killer cells during pregnancy. Similarly, increased α-defensin levels and monocyte and polymorphonuclear-cell activities suggest that progesterone is unfavorable for controlling MTB infection (Motomura et al., 2023). Emricasan, a pan-caspase inhibitor, suppresses enhanced apoptosis and inflammation. In non-alcoholic steatohepatitis, emricasan reduces hepatic inflammation and cirrhosis with good tolerance (Frenette et al., 2021). In a mouse model, emricasan and emricasan+doxycycline alleviated the lesion size and bacterial burden, indicating its potential as a host-directed immunotherapeutic against MRSA skin infections (Cahill et al., 2023). Emricasan has protective effects against Zika and SARS-CoV-2 infections (Alam et al., 2017; Plassmeyer et al., 2022).

Gene methylation regulates mRNA processing, affecting its stability, translocation, alternative splicing, and translation, therefore influencing various cellular processes. The m6A and m5C are two major RNA methylations that contribute to pulmonary TB progression (Zhao et al., 2017). For example, METTL3 transcription levels (a methyltransferase as an S-adenosylmethionine-binding subunit), METTL14 (RNA-binding scaffold for substrate recognition), and WTAP (interacts and localize METTL3 and METTL14 in nuclear speckles) have been found to substantially decreased in ATB patients (Zhang et al., 2022). Additionally, METTL3 and WTAP genetic variations were linked with TB susceptibility, suggesting their probable involvement in TB pathogenesis (Zhang et al., 2022). In the rat-injured kidney model, the ATF3 gene is regulated by METTL3 and WTAP meditated m6A methylation, promoting cell apoptosis (Li et al., 2021a). Li et al. (Li et al., 2022) found markedly decreased mRNA levels of YTHDF1, YTHDC1, and YTHDC2 in ATB individuals’ PBMC, consistent with this research. YTHDC2 variants were related to fever or sputum smear-positive in ATB patients and can elicit apoptosis by elevating caspase levels (Li et al., 2022). A previous study revealed that in HIV-1 co-infected MTB patients, DNA methyltransferase 1 (DNMT1) was markedly decreased (Marimani et al., 2020). *In-vitro* DNMT1 knockdown induces many genes, including ATF-3, and enhances apoptosis (Milutinovic et al., 2003). Consistent with this investigation, these m6A/m5C genes might be involved with TB pathogenesis by methylation modification of some ARG.

The major strength of this study is the combinational use of multiple bioinformatic analyses. For example, WGCNA offers multiple advantages over other bioinformatics methods because the analysis focuses on the link between clinical features and co-expression modules. By combining it with the PPI network for key genes, the results had high reliability and biological significance. Additionally, unlike previous studies, which screened gene signatures not limited to particular biological processes, this study was focused on the apoptosis-related genes as biomarkers to distinguish between ATB and LTBI, thereby providing evidence for their potential role in the pathogenesis of Mtb infection.

This study has some limitations. 1) Although all publicly accessible datasets were assessed, the sample size was relatively small, which might have interfered with conclusions. 2) The data on the association between immune cells, ARGs, and m6A/m5C methylation should be considered statistical, not causative. 3) Whether these host variables are exclusive to MTB infection is unclear. 4) Microarrays have multiple disadvantages (Not a whole genome analysis, higher background signal levels, not quantitative, unable to detect alternative splicing). To assess the involvement of these ARGs’ activity and underlying pathogenesis of pediatric ATB advanced from LTBI, further *in-vitro* and *in-vivo* investigations are required.

## 5 Conclusions

This study identified six ARGs (*CASP1, TNFSF10, CASP4, CASP5, IFI16*, and *ATF3*) that could be reliable biomarkers for differentiating ATB from LTBI. These ARGs may potentially participate in the immunopathogenesis of MTB infection by modifying m6A/m5C methylation. Agents targeting these ARGs that could be used as promising drug candidates for TB treatment were also screened. These findings highlight evidence for future research on MTB infection pathogenesis.

## Data availability statement

The datasets presented in this study can be found in online repositories. The names of the repository/repositories and accession number(s) can be found in the article/**Supplementary Material**.

## Ethics statement

The Ethics Committee of Nanjing Lishui People’s Hospital approved this study (No.2022SQ009), and the written informed contents were obtained from the subjects.

## Author contributions

XD: Conceptualization, Formal analysis, Data curation, Methodology Software, Supervision, Validation, Visualization. Writing – original draft. LZ: Data curation, Supervision, Validation, Writing – review & editing. XH: Data curation, Validation, Writing – review & editing. JH: Conceptualization, Data curation, Writing – review & editing. LC: Conceptualization, Data curation, Formal analysis, Funding acquisition, Investigation, Methodology, Project administration, Resources, Software, Supervision, Validation, Visualization, Writing – original draft, Writing – review & editing. YYL: Conceptualization, Data curation, Funding acquisition, Investigation, Methodology, Project administration; Resources, Software, Supervision, Validation, Visualization, Writing – review & editing.
